# Anti-inflammatory Effects of Homotaurine in Patients With Amnestic Mild Cognitive Impairment

**DOI:** 10.3389/fnagi.2018.00285

**Published:** 2018-11-02

**Authors:** Paola Bossù, Francesca Salani, Antonio Ciaramella, Eleonora Sacchinelli, Alessandra Mosca, Nerisa Banaj, Francesca Assogna, Maria Donata Orfei, Carlo Caltagirone, Walter Gianni, Gianfranco Spalletta

**Affiliations:** ^1^Laboratory of Experimental Neuropsychobiology, Department of Clinical and Behavioral Neurology, IRCCS Santa Lucia Foundation, Rome, Italy; ^2^Laboratory of Neuropsychiatry, Department of Clinical and Behavioral Neurology, IRCCS Santa Lucia Foundation, Rome, Italy; ^3^Laboratory of Psychology and Pediatric Pharmacology, Department of Neuroscience, University of Florence, Florence, Italy; ^4^Molecular Neurology Unit, CeSI-MeT, Center for Excellence on Aging and Translational Medicine, G. d’Annunzio University Chieti-Pescara, Chieti, Italy; ^5^Centro Fermi-Museo Storico della Fisica e Centro Studi e Ricerche Enrico Ferm, Rome, Italy; ^6^Department of Medicine of Systems, Tor Vergata University of Rome, Rome, Italy; ^7^Clinical and Behavioral Neurology, IRCCS Santa Lucia Foundation, Rome, Italy; ^8^II Clinica Medica, Sapienza University of Rome, Rome, Italy; ^9^Menninger Department of Psychiatry and Behavioral Sciences, Baylor College of Medicine, Houston, TX, United States

**Keywords:** tramiprosate, amnesic MCI, Alzheimer, APOE, inflammation, cytokines, interleukin-18

## Abstract

Alzheimer’s disease (AD) is a fatal dementing neurodegenerative disease, currently lacking an efficacious disease-modifying therapy. In the last years, there has been some interest in the use of homotaurine as a potential therapeutic compound for AD, but more work is still needed to prove its efficacy as disease modifier in dementia. Since inflammation is believed to play a key role in AD development, we sought to investigate here the *in vivo* homotaurine effect on inflammatory response in patients at the earliest stages of AD, i.e., suffering from amnestic mild cognitive impairment (aMCI). Thus, the present study aims to evaluate the effects of homotaurine supplementation on cytokine serum levels and memory performances in MCI patients. Neuropsychological, clinical and cytokine assessment was performed at baseline (T0) and after 1 year (T12) of homotaurine supplementation in 20 patients categorized as carriers (*n* = 9) or no carriers (*n* = 11) of the ε4 allele of the apolipoprotein E (APOE) gene, the strongest genetic risk factor for AD. The serum levels of the pro-inflammatory mediators Interleukin (IL) 1β, Tumor necrosis factor-alpha (TNFα), IL-6 and IL-18, contextually with the anti-inflammatory molecules IL-18 binding protein (IL-18BP) and Transforming growth factor-beta (TGFβ), were analyzed to explore significant differences in the inflammatory status between T0 and T12 in the two APOE variant carrier groups. No significant differences over time were observed in patients as for most cytokines, except for IL-18. Following homotaurine supplementation, patients carrying the APOEε4 allele showed a significant decrease in IL-18 (both in its total and IL-18BP unbound forms), in turn associated with improved short-term episodic memory performance as measured by the recency effect of the Rey 15-word list learning test immediate recall. Thus, homotaurine supplementation in individuals with aMCI may have a positive consequence on episodic memory loss due, at least in part, to homotaurine anti-inflammatory effects. This study strongly suggests that future research should focus on exploring the mechanisms by which homotaurine controls brain inflammation during AD progression.

## Introduction

Alzheimer’s disease (AD) is a neurodegenerative disorder characterized by progressive deterioration in cognition, functional ability and behavior, but its underlying causes are to date unclear and a disease-modifying cure is still unavailable. Dysfunction in the clearance of Amyloid β-protein (Aβ) and neuroinflammation are two functionally linked pathological aspects of key importance in the development and progression of the disease (Heppner et al., 2015).

Homotaurine is an aminosulfonate compound naturally found in red algae, which has been demonstrated to have neuroprotective effects in rats systemically administered with kainic acid (Fariello et al., 1982) or following ischemic stroke (Wu et al., 2014). As a possible therapeutic agent for AD treatment, homotaurine reduces Aβ levels in CSF of patients with mild to-moderate disease (Aisen et al., 2006), slows brain atrophy (Gauthier et al., 2009) and exerts positive effect on cognitive impairment (Saumier et al., 2009). In a phase III AD clinical trial, homotaurine does not satisfy pre-fixed primary outcomes failing to demonstrate changes in cognitive function compared to placebo, but this result is possibly confounded by high statistical variability of data and is paralleled by *post hoc* analyses in a subgroup of patients, revealing some protective effects on hippocampal volume loss (Aisen et al., 2011). Thus, although safe and well tolerated, homotaurine is not authorized as a new AD drug, but it is currently used as a nutraceutical for memory protection and its use in treatment of cognitive decline symptoms is still considered promising. According with its potential favorable effects, we recently demonstrated that homotaurine supplementation has a positive consequence on hippocampus atrophy and short-term episodic memory loss in individuals at the earliest clinical state of AD, namely subjects suffering from amnestic mild cognitive impairment (aMCI; Spalletta et al., 2016). Regardless the favorable disease-modifying activities of homotaurine, its therapeutic efficacy and mechanism of action have yet to be fully elucidated.

Intriguingly, the protective activity of homotaurine appears to be especially evident in AD patients carrying the apolipoprotein E (APOE) ε4 alleles (Caltagirone et al., 2012), suggesting that its effects might be influenced by APOE ε4 genotype, the most powerful genetic risk factor of AD. Since APOE proteins appear to modulate Aβ clearance (Kim et al., 2009) and from *in vitro* and preclinical studies homotaurine reduces soluble levels of Aβ, inhibits its aggregation and decreases its toxic effects on neurons (Gervais et al., 2007), it is tempting to speculate that homotaurine may act, at least in part, in an APOE-dependent way. Furthermore, since Aβ clearance defect might also be both cause and consequence of the chronically activated neuroinflammatory pathways, which in turn concur to cause neuronal death, we addressed this study to evaluate the ability of homotaurine supplementation in modulating the inflammatory response in treated aMCI patients. In fact, several studies indicate that cerebral Aβ deposits elicit a chronic, disseminated inflammatory response producing neurodegeneration in AD (Akiyama et al., 2000) and, more recently, a skewed immune response both in brain and periphery has been blamed for a defective Aβ clearance leading to AD development (Heneka et al., 2015; Marsh et al., 2016; Ransohoff, 2016). In this regard, accumulation of reactive (and possibly functionally flawed) microglia in damaged brain regions and increased cerebral/peripheral expression of pro-inflammatory cytokines have been broadly described in AD patients. Of note, in response to a peripheral inflammatory stimulus, pro-inflammatory cytokine production is higher with APOE ε4 genotype, compared to the other APOE allele, and recent observations suggest a role for APOE in modulating Aβ-induced neuroinflammation (Tai et al., 2015), supporting the relevance of APOE genotype-specific homotaurine therapeutic potential.

By a mechanistic point of view, Aβ may trigger an innate response through the activation of NALP3 inflammasome (Halle et al., 2008), a multi-protein innate immune pathogen-sensing complex, which is essential for the release of specific inflammatory mediators, determining the cytokine milieu in the brain. Indeed, the two inflammasome-dependent molecules Interleukin (IL)-1β and IL-18 have been shown to be crucial regulators in AD pathology (Shaftel et al., 2008; Bossù et al., 2010). These two cytokines may exert their inflammatory action at both brain and peripheral level. While circulating IL-1β is generally low in normal conditions, with serum concentration often below level of detection and very small visible effects in the periphery, serum IL-18 is constitutively present in high amounts but is regulated by a highly specific natural inhibitor, named IL-18 binding protein (IL-18BP).

Therefore, with the aim to evaluate the potential immunomodulating effects of homotaurine in the serum of APOE genotyped aMCI patients, in this study we measured a panel of pro- and anti-inflammatory cytokines, including other than IL-1β, Tumor necrosis factor-alpha (TNFα), IL-6 and Transforming growth factor-beta (TGFβ), also IL-18BP and IL-18. The latter was evaluated in both its forms, i.e., the total form, including the cytokine bound and unbound to its inhibitor, and the free, unbound and biologically active form. Because of previous evidence that the compound is effective on patients carrying the APOEε4 allele, we hypothesized that homotaurine has an effect on memory deficit and immunomodulation in this subgroup only.

## Materials and Methods

### Subjects

Twenty subjects with aMCI were included in this study. Only patients without significant clinical factors that promote inflammation were selected. Particular attention was given to exclude patients suffering from those diseases that are known to be associated with altered cytokine production that may be common in elderly people, such as autoimmune diseases (e.g., Crohn’s disease, rheumatoid arthritis, systemic lupus erythematosus), infections and cancer. Nine of the 20 aMCI patients were carriers of the APOEε4 allele (including eight patients with ε3/ε4 and only one with ε4/ε4 APOE genotype) and 11 were no carriers (including 10 patients with ε3/ε3 and 1 with ε2/ε3 APOE genotype).

A trained senior research psychiatrist (GS) made the diagnosis of aMCI and two trained post-doc neuropsychologists made the cognitive assessment. All the clinical evaluation and blood sampling were performed at the baseline and after 12 months of supplementation with homotaurine. Medical and psychiatric histories were obtained from each subject, and they all underwent a series of standard clinical examinations, including physical, neurological and mental status examinations, neurocognitive tests, and brain magnetic resonance imaging. No patient had taken antidementia drugs lifetime, or psychotropic drugs (i.e., antidepressants, benzodiazepines and antipsychotics) in the previous 12 months.

Inclusion criteria for aMCI were: (1) diagnostic evidence of amnestic MCI consistent with Petersen guidelines (Petersen et al., 1997) and (2) a Mini Mental State Examination (MMSE) score ≥23. In particular, for the diagnosis of aMCI was required impaired performance on at least one memory test in association or not with impaired performance in at least one additional cognitive domain (i.e., praxis, attention, language and executive functions) in the absence of functional impairment. Exclusion criteria were: (1) major medical illnesses and autoimmune-inflammatory diseases; (2) co morbidity of primary psychiatric or neurological disorders and any other significant mental or neurological disorder; (3) clinically important infection within the last 30 days (e.g., chronic persistent or acute infection, such as bronchitis or urinary tract infection); (4) implant of carotid or coronary stent or other major surgical interventions; (5) use of anti-inflammatory drugs within the last 60 days (e.g., corticosteroids or nonsteroidal anti-inflammatory drugs). (6) MRI evidence of focal parenchymal abnormalities or neoplasm. The included aMCI patients underwent the first diagnostic assessment for memory problems in the Santa Lucia Foundation outpatient memory clinic in Rome and were treated with homotaurine tablets, 50 mg, QD for 2 weeks and BID for the next year. Informed written consent was obtained from all subjects or, when necessary, from their proxies in accordance with the Declaration of Helsinki. The protocol was approved by the “Fondazione Santa Lucia Ethics committee.” Demographic and clinical characteristics of subjects included in the study are summarized in Table 1.

**Table 1:** Sociodemographic and clinical characteristics at the baseline in amnestic mild cognitive impairment (aMCI) patients treated with homotaurine carriers and no carriers of the apolipoprotein E (APOE) ε4 allele.

	Individuals				
NO ε4	ε4			
Characteristics	*n* = 11; Mean ± SD	*n* = 9; Mean ± SD	T or chi-square	*df*	*p*
Age	71.14 ± 6.01	73.36 ± 7.46	−0.926	31	0.362
Educational level	9.77 ± 3.70	11.64 ± 4.30	−1.293	31	0.206
Gender male (*n*, %)	6 (55%)	5 (55%)	0	1	>0.999
MMSE score (baseline)	26.95 ± 2.08	26.82 ± 2.04	0.179	31	0.859
MMSE score (follow-up)	26.36 ± 2.4	25.98 ± 2.43	1.567	31	0.1279
IADL	6.82 ± 1.68	7.54 ± 1.92	−1.119	31	0.272

*SD, standard deviation; MMSE, Mini-Mental State Examination; IADL, instrumental activities of daily living*.

### Neuropsychological and Functional Assessment

The MMSE was administered to obtain a global index of cognitive impairment at the baseline and the Mental Deterioration Battery (MDB; Carlesimo et al., 1996) to measure performance in specific cognitive domains at baseline and the 1-year follow-up. In particular, the MDB was administered to make a comprehensive cognitive examination at the diagnostic level.

In order to measure episodic memory performance, Rey’s 15-word list learning test (RLT) was administered. In this task participants are given a list of 15 unrelated words that are repeated in five different trials and asked to recall them in any order immediately afterwards (immediate recall (I-RLT); score range: 0–75). After a 15-min interval, during which non-verbal tasks are given, the patient is asked to recall, without list repetition, as many words as possible in any order (delayed recall (D-RLT); score range: 0–15). The task allows characterizing patterns of performance relative to the position of the items in the word list, because recall accuracy varies as a function of the item’s position in the study list, i.e., it is greater for words at the beginning (primacy effect) and the end (recency effect) of the list compared to the mid-list (intermediate) items (Murdock, 1962). Special scoring of word-list recall data for serial position has been suggested to improve discrimination of normal aging from dementia. Specifically, the number of correctly recalled words in the early list positions (from word 1 to word 5), the intermediate list positions (from word 6 to word 9), and the number of correctly recalled recency items (from word 10 to word 15) was calculated. The latter effect is the most efficient measure of the short-term component of episodic memory as recency items are stored in a short-term phonological buffer and probably still present in working memory when recall is solicited (Vallar and Papagno, 1986).

Functional impairment was evaluated by assessing instrumental activities of daily living (IADL; Lawton and Brody, 1969). The IADL include abilities that allow a person to live independently (e.g., food preparation, housekeeping and laundry, managing financial matters, shopping and using a telephone). When the ability is fully or at least partially preserved a score of 1 is assigned; when the ability has been lost a score of 0 is assigned. Thus, a total score ranging from 0 (total dependance) to 8 (5 for men) for IADL (total independance) is obtained.

### Blood, APOE Genotyping and Cytokine Measurement

Blood from included aMCI subjects, who had been fasting for approximately 10 h, was drawn in the morning, both at the baseline (T0) and at 1-year follow-up from homotaurine supplementation (T12).

Genomic DNA was purified from whole blood drawn at baseline and the APOE genotyping assessed by real-time polymerase chain reaction (PCR) using the LightCycler ApoE Mutation Detection Kit (Roche Diagnostics).

Serum samples were obtained at both T0 and T12 time points by centrifugation of clotted blood and aliquots were stored at −80°C until cytokine assays. All serum cytokines were measured by commercially available enzyme-linked immunosorbent assay (ELISA), in accordance with the manufacturer’s instructions. Specifically, IL-1β, TNFα and IL-6 were evaluated by high sensitivity ELISA kits named Human Quantikine HS (R&D Systems, Minneapolis, MN, USA), with detection limits equivalent to 0.125, 0.5 and 0.156 pg/ml, respectively. TGFβ was measured by Human TGFβ1 cytoset kit (Invitrogen) and the detection limit of the assay was 31 pg/ml. IL-18 levels, corresponding to the total amount of the cytokine, i.e., both its forms: bound and not bound to its natural inhibitor IL-18BP, were determined using coating antibody (clone 125-2H), detecting antibody (clone 159-12B) and standard human recombinant IL-18 (MBL, Nagoya, Japan). IL-18BP levels were measured by an ELISA kit specifically recognizing IL-18BPa, the prevalent isoform in humans (R&D Systems, Minneapolis, MN, USA). Detection limit for both IL-18 and IL-18BP assays was 12.5 pg/mL. Concentrations of free IL-18, namely bioactive IL-18 unbound to its inhibitor, resulted from calculation based on the law of mass action, considering 1:1 stoichiometry in the complex of IL-18 and IL-18BP and a dissociation constant (Kd) of 0.4 nM, as elsewhere reported in more detail (Migliorini et al., 2010).

### Statistical Analysis

**Figure 1:**
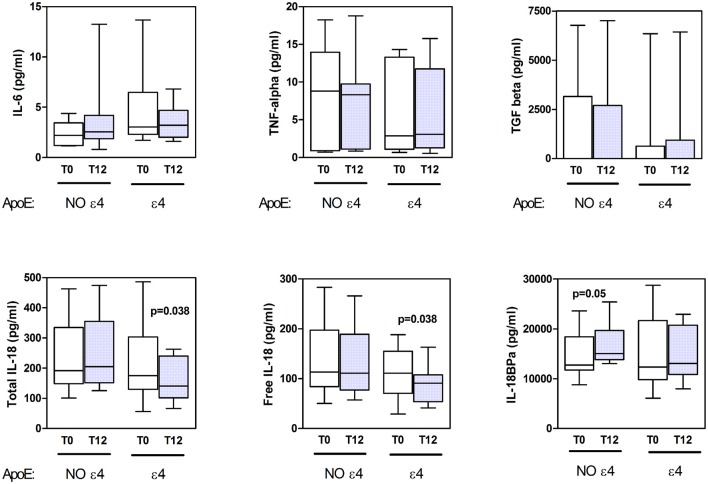
Cytokine serum levels. The concentration of serum cytokines in amnestic mild cognitive impairment (aMCI) subjects genotyped as apolipoprotein E (Apo)Eε4 no carriers (NO ε4) and ApoEε4 carriers (ε4) at the baseline (T0) and after 12 months (T12) from homotaurine supplementation are reported in the respective box-plot displaying the distribution of data and median values, as indicated.

StatView (SAS, San Francisco, CA, USA) and GraphPad (Prism Version 4, San Diego, CA, USA) software were used for statistical analyses. Because the data do not follow normal distribution (as showed in Figure 1), non-parametric statistical tests were used. In particular, the differences between continuous variables at baseline and after 12 months of homotaurine supplementation were evaluated using Wilcoxon Signed Ranks test. Kendall Rank Correlation assessed relationship between IL-18 values and memory (i.e., I-RLT primacy and recency effects) performances. *P*-values less than 0.05 were considered statistically significant.

## Results

### Reduced Serum IL-18 Levels After Homotaurine Supplementation in MCI Patients Carrying the APOE ε4 Allele

The cytokine serum levels of aMCI subjects, as evaluated at the baseline and after 12 months from homotaurine supplementation, are illustrated in Figure 1. Given the potential impact of ApoE genotype on inflammatory status, patients were categorized as APOE ε4 carriers (*n* = 9) and no APOE ε4 carriers (*n* = 11) and cytokine levels were evaluated accordingly, both at baseline and 1-year follow-up.

Regardless of some apparent fluctuations, especially about IL-6, there were not significant changes between baseline and follow-up levels regarding IL-6, TNFα and TGFβ, both in ε4 carriers and no carriers patients (Figure 1 upper panels).

Notably, among all cytokines tested, only IL-18 levels resulted significantly different between before and after homotaurine supplementation. In fact, aMCI patients carrying the APOE ε4 genotype showed reduced levels of both total and free (biologically active) IL-18 after 12 months of homotaurine supplementation, as compared to baseline (Figure 1, lower left and lower central panels, respectively). This decrease was not paralleled by changes in the IL-18BP levels. At variance, patients not carrying the APOE ε4 allele exhibited a slight increase of the IL-18 inhibitor following the homotaurine supplementation (Figure 1, lower right panel), with values approaching the statistical significance.

The levels of IL-1β were always below the sensitivity of our assay and therefore undetectable in all patients (not shown).

### Correlation Between Serum IL-18 Levels and Memory Performance Scores (Recency Effect) in MCI Patients

**Figure 2:**
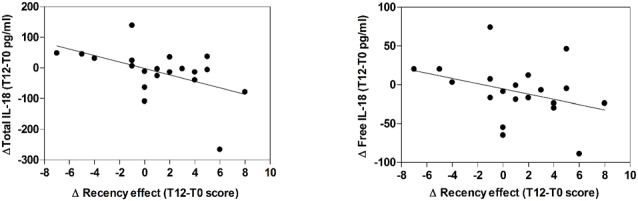
Correlation between serum IL-18 levels and memory performance scores. The relationship between T12-T0 delta values of Total (right panel) or Free (left panel) IL-18 levels and recency effect score is reported in the respective scatterplot, as indicated. Lines represent linear regression.

Values of Primacy and recency effects of the I-RLT are reported in Table 2. Delta scores (considered as follow-up values minus baseline values) were calculated for the primacy and recency effects of the I-RLT and for the IL-18 cytokine. The Kendall Rank correlation analysis indicated a significant relationship (Tau = −0.395; Tied Z-value = −2.507; *p* = 0.012) between delta values of total IL-18 levels and recency effect score. In addition, results approached the significance (Tau = −0.300; Tied Z-value −1.915; *p* = 0.055) for relationship between delta values of free IL-18 levels and recency effect score. In both cases, decreased cytokine level over the supplementation therapy was correlated with increased memory performance (Figure 2).

**Table 2:** Primacy, Intermediate and Recency effects in aMCI-APOE ε4 carriers and no carriers at the baseline and the 1-year follow-up.

Episodic memory effects	Baseline		T0 vs. T0	1-Year		Wilcoxon	Delta (T1-T0)		Effect of group on delta
	NO ε4 (*n* = 11)	ε4 (*n* = 9)		NO ε4 (*n* = 11)	ε4 (*n* = 9)		NO ε4 (*n* = 11)	ε4 (*n* = 9)
	(Mean ± SD)	(Mean ± SD)	*t*; *p*	(Mean ± SD)	(Mean ± SD)	*F*; *p*	(Mean ± SD)	(Mean ± SD)	U; *p*
Primacy effect (I-RWLLT)	8.41 ± 3.25	10.73 ± 3.85	−1.82; 0.08	8.50 ± 4.86	7.91 ± 3.59	3.23; 0.08	0.09 ± 4.35	−2.81 ± 4.44	168; 0.07
Intermediate effect (I-RWLLT)	3.64 ± 2.42	4.45 ± 2.98	−0.85; 0.40	4.45 ± 3.11	4.64 ± 2.98	0.50; 0.49	0.82 ± 2.59	0.18 ± 2.18	133; 0.65
Recency effect (I-RWLLT)	10.04 ± 3.26	11.18 ± 3.43	−0.93; 0.36	9.04 ± 3.48	13.18 ± 3.89	5.01; 0.03	−1 ± 3.59	2 ± 3.71	181.5; 0.02

*SD, standard deviation; I-RWLLT, Rey 15-word list learning test immediate recall*.

## Discussion

The main outcome of this study reveals that in aMCI patients, who carry the APOE ε4 allele, a 12-month supplementation with homotaurine is specifically associated with a decrease in the serum levels of the pro-inflammatory cytokine IL-18, in turn related to improved episodic memory performances. This finding corroborates previous data showing that the Aβ aggregation-preventing compound homotaurine exerts protective effects on AD in conditions of APOE ε4 genotype (Caltagirone et al., 2012) and in the early stages of the disease, as holding a positive effect on hippocampus atrophy and short-term episodic memory loss in amnestic MCI patients (Spalletta et al., 2016). Of importance, the present work offers novel evidence on homotaurine anti-inflammatory potential in the very precocious clinical phases of aMCI.

Indeed, neuroinflammation is involved early in the pathogenesis of AD neurodegeneration by taking part in a vicious cycle of Aβ deposition, neuronal death and cognitive decline (Eikelenboom et al., 2010; Heppner et al., 2015). Pro-inflammatory cytokines, including those released into the brain as well as in the periphery, like IL-18, are able to modulate the activation of resident microglia and participate in neuronal degeneration through different mechanisms. An increase in circulating pro-inflammatory factors, including IL-18, has been often observed in patients, as compared to healthy controls (Yaffe et al., 2003; Öztürk et al., 2007; Swardfager et al., 2010; Trollor et al., 2010; Brosseron et al., 2014; Saleem et al., 2015). However, several reports have described controversial results on cytokine blood levels in AD or MCI, mainly because of high inter-individual variability, clinical confounders and differences in assay procedures. Thus, if on the one hand, these observations confirm the potential of circulating inflammatory mediators as index of disease, on the other hand they encourage performing further longitudinal studies that may clarify the issue. In fact, despite the fact we did not observe any difference of serum cytokine levels between baseline and 12 months supplementation with homotaurine in our whole group of aMCI, we found the intriguing result when we split the group based on APOE genotype, according with our hypothesis. Such result confirms the need to limit heterogeneity among patients to identify potential cytokine markers of disease.

Noteworthy, the only over time change in aMCI subgroups is IL-18, making this cytokine an inflammatory mediator of special interest not only in association with homotaurine supplementation but, possibly, in the overall MCI context. In addition, since IL-18 herein exemplifies an inflammasome-dependent cytokine (unfortunately serum levels of IL-1β were undetectable), our results suggest that the activation of the inflammasome complex may play an important role in homotaurine action and conceivably in neurodegenerative dementia progression, as indeed recently reported (Saresella et al., 2016).

Furthermore, the observed homotaurine supplementation-dependent reduction of IL-18 concerned the two circulating forms of IL-18: both total and free, IL-18BP-unbound cytokine, with no effects on the inhibitory molecule IL-18BP. Differently, a trend towards IL-18BP increase was observed in treated MCI not carrying the APOE ε4 allele, suggesting that homotaurine plays a different regulation of IL-18, depending on the APOE genotype. The latter is a quite intriguing result, which strongly encourage driving additional research efforts to the comprehension of the interconnected molecular pathways linking APOE and neuroinflammation to the increased risk to develop AD, as well as to the potential effects played by homotaurine in this context. In fact, as previously reported, ApoE isoforms affect inflammatory processes (Vitek et al., 2009), with APOE ε4 carriers having lower brain levels of ApoE protein, enhanced neuroinflammation, and greater accumulation of Aβ. Hence, the AD-linked inflammatory events may be influenced by the function of ApoE proteins, giving rise to an interesting interpretation that Aβ-dependent inflammation may participate in neurodegenerative processes involving ApoE-mediated mechanisms (Tai et al., 2015). In particular, since Aβ-dependent triggering of inflammation involves the activation of both the transcription factor Nuclear Factor kappa B (NF-kB) and NLRP3 inflammasome (Halle et al., 2008; Vallabhapurapu and Karin, 2009), which together lead to IL-18 production, it is possible that homotaurine, by lowering Aβ levels can also negatively modulate Aβ-dependent NF-kB signaling, inflammasome activation and, eventually, IL-18 release.

The second achievement of this study points to an association between the homotaurine-dependent decrease of serum IL-18 and the improvement of short-term episodic memory performances. Although it remains unclear whether IL-18 plays a role (either primary or secondary) in neurodegeneration-associated cognitive decline, the observed decrease of serum IL-18 after homotaurine supplementation and its significant correlation with clinical parameters sustain the pathogenic importance of IL-18 in AD. Our result complements a whole body of literature describing that IL-18 might inhibit the cellular mechanisms underlying learning and memory (Curran and O’Connor, 2001) and participate in different ways to neuronal damage (Alboni et al., 2010), proposing that this pro-inflammatory cytokine and its related molecular pathways are of some relevance in AD pathogenesis (Bossù et al., 2010).

The present piece of work suggests that homotaurine supplementation in aMCI individuals carrying APOE ε4 has a positive consequence on episodic memory loss due, at least in part, to homotaurine anti-inflammatory effects targeting IL-18. While some studies highlighted the potential of homotaurine and its derivatives to benefit patients with MCI (Martorana et al., 2014; Spalletta et al., 2016), and to modulate inflammation in mouse models (Sternberg et al., 2012), this is the first evidence ever provided that homotaurine supplementation holds anti-inflammatory properties associated with memory improvement in patients with cognitive impairment.

This finding sheds a new light on the therapeutic potential of homotaurine, sustaining its beneficial disease-modifying effects, encouraging further confirmatory studies for treating genetically-defined populations with moderate AD (Abushakra et al., 2017), and also inspiring future research aimed at exploring the mechanisms by which this compound might control brain inflammation during progression of neurodegenerative dementias.

## Author Contributions

PB and GS planned and supervisioned the study. FS, AC, ES and AM designed and performed experiments. NB, FA, MO, CC, WG and GS coordinated and performed clinical evaluations. PB, GS, FS, AC, FA and MO analyzed the data. PB and GS wrote the article. All authors made substantial contributions to the conception of the work and its interpretation of data. All authors critically revised the manuscript and approved its finalversion.

## Conflict of Interest Statement

Some reagents used for this study have been provided by FB Health, in complete absence of any influence from FB Health on the submitted work. Consulting Fees GS was funded from FB Health and Novartis for consultancy outside the submitted work. Lecture Fees GS received one lecture fee from FB Health. The remaining authors declare that the research was conducted in the absence of any commercial or financial relationships that could be construed as a potential conflict of interest.

## References

[B1] Abushakra S. Porsteinsson A. Scheltens P. Sadowsky C. Vellas B. Cummings J.. (2017). Clinical effects of tramiprosate in APOE4/4 homozygous patients with mild Alzheimer’s disease suggest disease modification potential. J. Prev. Alzheimers Dis. 4, 149–156. 10.14283/jpad.2017.26 29182706

[B2] Aisen P. S. Gauthier S. Ferris S. H. Saumier D. Haine D. Garceau D.. (2011). Tramiprosate in mild-to-moderate Alzheimer’s disease—a randomized, double-blind, placebo-controlled, multi-centre study (the Alphase Study). Arch. Med. Sci. 7, 102–111. 10.5114/aoms.2011.20612 22291741 PMC3258678

[B3] Aisen P. S. Saumier D. Briand R. Laurin J. Gervais F. Tremblay P.. (2006). A Phase II study targeting amyloid-β with 3APS in mild-to-moderate Alzheimer disease. Neurology 67, 1757–1763. 10.1212/01.WNL.0000244346.08950.64 17082468

[B4] Akiyama H. Barger S. Barnum S. Bradt B. Bauer J. Cole G. M.. (2000). Inflammation and Alzheimer’s disease. Neurobiol. Aging 21, 383–421. 10.1016/S0197-4580(00)00124-X 10858586 PMC3887148

[B5] Alboni S. Cervia D. Sugama S. Conti B. (2010). Interleukin 18 in the CNS. J. Neuroinflammation 7:9. 10.1186/1742-2094-7-9 20113500 PMC2830964

[B6] Bossù P. Ciaramella A. Salani F. Vanni D. Palladino I. Caltagirone C.. (2010). Interleukin-18, from neuroinflammation to Alzheimer’s disease. Curr. Pharm. Des. 16, 4213–4224. 10.2174/138161210794519147 21184660

[B7] Brosseron F. Krauthausen M. Kummer M. Heneka M. T. (2014). Body fluid cytokine levels in mild cognitive impairment and Alzheimer’s disease: a comparative overview. Mol. Neurobiol. 50, 534–544. 10.1007/s12035-014-8657-1 24567119 PMC4182618

[B8] Caltagirone C. Ferrannini L. Marchionni N. Nappi G. Scapagnini G. Trabucchi M. (2012). The potential protective effect of tramiprosate (homotaurine) against Alzheimer’s disease: a review. Aging Clin. Exp. Res. 24, 580–587. 10.3275/8585 22961121

[B9] Carlesimo G. A. Caltagirone C. Gainotti G. (1996). The mental deterioration battery: normative data, diagnostic reliability and qualitative analyses of cognitive impairment. The group for the standardization of the mental deterioration battery. Eur. Neurol. 36, 378–384. 10.1159/000117297 8954307

[B10] Curran B. O’Connor J. (2001). The pro-inflammatory cytokine interleukin-18 impairs long-term potentiation and NMDA receptor-mediated transmission in the rat hippocampus *in vitro*. Neuroscience 108, 83–90. 10.1016/s0306-4522(01)00405-5 11738133

[B11] Eikelenboom P. van Exel E. Hoozemans J. J. M. Veerhuis R. Rozemuller A. J. M. van Gool W. A. (2010). Neuroinflammation—An early event in both the history and pathogenesis of Alzheimer’s disease. Neurodegener. Dis. 7, 38–41. 10.1159/000283480 20160456

[B12] Fariello R. G. Golden G. T. Pisa M. (1982). Homotaurine (3 aminopropanesulfonic acid; 3APS) protects from the convulsant and cytotoxic effect of systemically administered kainic acid. Neurology 32, 241–245. 10.1212/wnl.32.3.241 7199633

[B13] GauthierS.AisenP. S.FerrisS. H.SaumierD.DuongA.HaineD.. (2009). Effect of tramiprosate in patients with mild-to-moderate Alzheimer’s disease: exploratory analyses of the MRI sub-group of the Alphase study. J. Nutr. Heal. Aging 13, 550–557. 10.1007/s12603-009-0106-x19536424

[B14] Gervais F. Paquette J. Morissette C. Krzywkowski P. Yu M. Azzi M.. (2007). Targeting soluble Aβ peptide with Tramiprosate for the treatment of brain amyloidosis. Neurobiol. Aging 28, 537–547. 10.1016/j.neurobiolaging.2006.02.015 16675063

[B15] Halle A. Hornung V. Petzold G. C. Stewart C. R. Monks B. G. Reinheckel T.. (2008). The NALP3 inflammasome is involved in the innate immune response to amyloid-β. Nat. Immunol. 9, 857–865. 10.1038/ni.1636 18604209 PMC3101478

[B16] Heneka M. T. Carson M. J. El Khoury J. Landreth G. E. Brosseron F. Feinstein D. L.. (2015). Neuroinflammation in Alzheimer’s disease. Lancet Neurol. 14, 388–405. 10.1016/S1474-4422(15)70016-5 25792098 PMC5909703

[B17] Heppner F. L. Ransohoff R. M. Becher B. (2015). Immune attack: the role of inflammation in Alzheimer disease. Nat. Rev. Neurosci. 16, 358–372. 10.1038/nrn3880 25991443

[B18] Kim J. Basak J. M. Holtzman D. M. (2009). The role of apolipoprotein E in Alzheimer’s disease. Neuron 63, 287–303. 10.1016/j.neuron.2009.06.026 19679070 PMC3044446

[B19] Lawton M. P. Brody E. M. (1969). Assessment of older people: self-maintaining and instrumental activities of daily living. Gerontologist 9, 179–186. 10.1093/geront/9.3_part_1.179 5349366

[B20] Marsh S. E. Abud E. M. Lakatos A. Karimzadeh A. Yeung S. T. Davtyan H.. (2016). The adaptive immune system restrains Alzheimer’s disease pathogenesis by modulating microglial function. Proc. Natl. Acad. Sci. U S A 113, E1316–E1325. 10.1073/pnas.1525466113 26884167 PMC4780638

[B21] Martorana A. Di Lorenzo F. Manenti G. Semprini R. Koch G. (2014). Homotaurine induces measurable changes of short latency afferent inhibition in a group of mild cognitive impairment individuals. Front. Aging Neurosci. 6:254. 10.3389/fnagi.2014.00254 25295005 PMC4172065

[B22] Migliorini P. Anzilotti C. Pratesi F. Quattroni P. Bargagna M. Dinarello C. A.. (2010). Serum and urinary levels of IL-18 and its inhibitor IL-18BP in systemic lupus erythematosus. Eur. Cytokine Netw. 21, 264–271. 10.1684/ecn.2010.0210 21126942

[B23] Murdock B. B. J. (1962). The serial position effect of free recall. J. Exp. Psychol. 64, 482–488. 10.1037/h0045106

[B24] Öztürk C. Özge A. Yalin O. Ö. Yilmaz I. A. Delialioglu N. Yildiz Ç.. (2007). The diagnostic role of serum inflammatory and soluble proteins on dementia subtypes: correlation with cognitive and functional decline. Behav. Neurol. 18, 207–215. 10.1155/2007/432190 18430978 PMC5469966

[B25] Petersen R. C. Smith G. E. Waring S. C. Ivnik R. J. Kokmen E. Tangelos E. G. (1997). Aging, memory and mild cognitive impairment. Int. Psychogeriatr. 9, 65–69. 10.1017/S1041610297004717 9447429

[B26] Ransohoff R. M. (2016). How neuroinflammation contributes to neurodegeneration. Science 353, 777–783. 10.1126/science.aag2590 27540165

[B27] Saleem M. Herrmann N. Swardfager W. Eisen R. Lanctot K. L. (2015). Inflammatory markers in mild cognitive impairment: a meta-analysis. J. Alzheimers Dis. 47, 669–679. 10.3233/jad-150042 26401702

[B28] Saresella M. La Rosa F. Piancone F. Zoppis M. Marventano I. Calabrese E.. (2016). The NLRP3 and NLRP1 inflammasomes are activated in Alzheimer’s disease. Mol. Neurodegener. 11:23. 10.1186/s13024-016-0088-1 26939933 PMC4778358

[B29] SaumierD.DuongA.HaineD.GarceauD.SampalisJ. (2009). Domain-specific cognitive effects of tramiprosate in patients with mild to moderate Alzheimer’s disease: ADAS-cog subscale results from the alphase study. J. Nutr. Heal. Aging 13, 808–812. 10.1007/s12603-009-0217-419812871

[B30] Shaftel S. S. Griffin W. S. T. O’Banion M. K. (2008). The role of interleukin-1 in neuroinflammation and Alzheimer disease: an evolving perspective. J. Neuroinflammation 5:7. 10.1186/1742-2094-5-7 18302763 PMC2335091

[B31] Spalletta G. Cravello L. Gianni W. Piras F. Iorio M. Cacciari C.. (2016). Homotaurine effects on hippocampal volume loss and episodic memory in amnestic mild cognitive impairment. J. Alzheimers Dis. 50, 807–816. 10.3233/jad-150484 26757035

[B32] Sternberg Z. Cesario A. Rittenhouse-Olson K. Sobel R. A. Leung Y. K. Pankewycz O.. (2012). Acamprosate modulates experimental autoimmune encephalomyelitis. Inflammopharmacology 20, 39–48. 10.1007/s10787-011-0097-1 22090150

[B33] Swardfager W. Lanctt K. Rothenburg L. Wong A. Cappell J. Herrmann N. (2010). A meta-analysis of cytokines in Alzheimer’s disease. Biol. Psychiatry 68, 930–941. 10.1016/j.biopsych.2010.06.012 20692646

[B34] Tai L. M. Ghura S. Koster K. P. Liakaite V. Maienschein-Cline M. Kanabar P.. (2015). APOE -modulated Aβ-induced neuroinflammation in Alzheimer’s disease: current landscape, novel data and future perspective. J. Neurochem. 133, 465–488. 10.1111/jnc.13072 25689586 PMC4400246

[B35] Trollor J. N. Smith E. Baune B. T. Kochan N. A. Campbell L. Samaras K.. (2010). Systemic inflammation is associated with MCI and its subtypes: the Sydney memory and aging study. Dement. Geriatr. Cogn. Disord. 30, 569–578. 10.1159/000322092 21252552

[B36] Vallabhapurapu S. Karin M. (2009). Regulation and function of NF-κB transcription factors in the immune system. Annu. Rev. Immunol. 27, 693–733. 10.1146/annurev.immunol.021908.132641 19302050

[B37] Vallar G. Papagno C. (1986). Phonological short-term store and the nature of the recency effect: evidence from neuropsychology. Brain Cogn. 5, 428–442. 10.1016/0278-2626(86)90044-8 3580186

[B38] Vitek M. P. Brown C. M. Colton C. A. (2009). APOE genotype-specific differences in the innate immune response. Neurobiol. Aging 30, 1350–1360. 10.1016/j.neurobiolaging.2007.11.014 18155324 PMC2782461

[B39] Wu S. Yue Y. Tian H. Tao L. Wang Y. Xiang J.. (2014). Tramiprosate protects neurons against ischemic stroke by disrupting the interaction between PSD95 and nNOS. Neuropharmacology 83, 107–117. 10.1016/j.neuropharm.2014.04.010 24769446

[B40] Yaffe K. Lindquist K. Penninx B. W. Simonsick E. M. Pahor M. Kritchevsky S.. (2003). Inflammatory markers and cognition in well-functioning African-American and white elders. Neurology 61, 76–80. 10.1212/01.wnl.0000073620.42047.d7 12847160

